# Effect of Intestinal Levodopa-Carbidopa Infusion on Pharyngeal Dysphagia: Results from a Retrospective Pilot Study in Patients with Parkinson's Disease

**DOI:** 10.1155/2020/4260501

**Published:** 2020-03-11

**Authors:** Bendix Labeit, Inga Claus, Paul Muhle, Sonja Suntrup-Krueger, Rainer Dziewas, Tobias Warnecke

**Affiliations:** ^1^University Hospital Muenster, Department of Neurology with Institute for Translational Neurology, Albert-Schweitzer-Campus 1; Gebäude A1, Münster 48149, Germany; ^2^Institute of Biomagnetism and Biosignalanalysis, University Hospital Muenster, University of Muenster, Malmedyweg 15, Münster 48149, Germany

## Abstract

**Background:**

Pharyngeal dysphagia is a common symptom of Parkinson's disease (PD) leading to severe complications. PD-related pharyngeal dysphagia (PDrPD) may significantly improve in up to half of patients following acute oral levodopa challenge.

**Objective:**

The aim of this study was to investigate the effects of levodopa-carbidopa intestinal gel (LCIG) on PDrPD.

**Methods:**

Forty-five PD patients under LCIG treatment were available for retrospective analysis. In all patients with PDrPD who underwent flexible endoscopic evaluation of swallowing (FEES) in the clinical “on-state” both before and after implementation of LCIG treatment, FEES videos were systematically reassessed. PDrPD was characterized using a PD-specific FEES score evaluating premature bolus spillage, penetration/aspiration, and pharyngeal residue. Further, the duration of white-out was assessed, as a parameter for pharyngeal bradykinesia.

**Results:**

Eleven patients with PDrPD (mean age 74.6 ± 4.4 years; mean Hoehn and Yahr stage 3.8 ± 0.6) received FEES both before and after the onset of LCIG treatment. The mean swallowing score improved from 14.9 ± 7.3 to 13.0 ± 6.9 after implementation of LCIG; however, this difference was not significant (*p*=0.312). Premature bolus spillage decreased significantly (*p*=0.002) from 5.4 ± 1.1 to 3.6 ± 1.0, and white-out duration decreased significantly (*p*=0.002) from 984 ± 228 ms to 699 ± 131 ms after implementation of LCIG.

**Conclusions:**

LCIG may affect PDrPD and reduce premature bolus spillage and pharyngeal bradykinesia. Future studies with larger sample sizes are required to follow-up on these pilot results and identify which factors predict a good response of PDrPD to LCIG treatment.

## 1. Introduction

In the advanced stage of Parkinson's disease (PD), about 40% of patients experience motor fluctuations [1]. Frequently, it becomes challenging to treat these PD patients sufficiently by oral medication [2]. Here, levodopa-carbidopa intestinal gel (LCIG) treatment offers an alternative drug delivery route to counteract motor fluctuations: a mixture of levodopa/carbidopa gel is continuously injected into the proximal small intestine via percutaneous endoscopic gastrojejunostomy (PEG-J), thereby bypassing the upper gastrointestinal tract. This leads to a more stable plasma concentration of the volatile levodopa (L-dopa) [3, 4]. LCIG has proven to be an effective escalation therapy in patients with drug-associated motor fluctuations [5]: Significant decreases in “off-time” and increases in “on-time” responses were reported without troublesome dyskinesia [6].

Pharyngeal dysphagia is frequent in patients with PD [7] and causes severe complications in the advanced stage of the disease such as aspiration pneumonia, which is a leading cause of death in this population [8]. The pathophysiology of PD-related pharyngeal dysphagia (PDrPD) is complex and includes both central and peripheral mechanisms [9]. Dopamine deficiency appears to play an important role because about half of the patients with PDrPD respond to the acute oral levodopa challenge [10]. For the assessment of PDrPD, instrumental procedures such as flexible endoscopic evaluation of swallowing (FEES) are recommended as the gold standard, as clinical evaluation is unable to reliably detect all relevant symptoms, e.g., silent aspiration [11, 12].

Despite its clinical importance, the impact of LCIG treatment on PDrPD has not yet been investigated with instrumental tools. Therefore, the aim of this study was to evaluate the effect of LCIG on PDrPD using FEES videos.

## 2. Methods

The study protocol was approved by the local ethics committee at the University of Muenster (2019-277-f-S). As the design was completely retrospective, the ethics committee waived the need for informed consent of individual patients.

### 2.1. Patient Cohort

This retrospective study included patients with PD treated at University Hospital Muenster that had received LCIG therapy between 01/2012 and 11/2018. During this period, LCIG therapy was generally applied when substantial daily motor fluctuations and dyskinesia were present that could not be sufficiently controlled with oral PD medication depending on the patient's preference and contraindications for other escalation therapies. Idiopathic PD was diagnosed according to the British Parkinson's Society Brain Bank criteria [13]. Patients with other pre-existing disease conditions associated with dysphagia (e.g., stroke) were excluded from this study. In a second step for final selection, patients were only included for further analysis if they had received a FEES examination that showed signs for PDrPD before the beginning of LCIG and a follow-up FEES after implementation of LCIG therapy in the clinical on-state. Signs for PDrPD were defined as either penetration/aspiration or at least mild pharyngeal residue according to the Yale Pharyngeal Residue Scale [14] or premature bolus spillage into the piriform sinus.

### 2.2. Clinical Parameters

Gender, age, time of disease, time of LCIG, signs for dysphagia according to FEES, dietary supplements, infections, pneumonia, discontinuation of LCIG, complication of LCIG, dislocation of LCIG, death, and signs for dementia were assessed by the patient chart review in December 2018. In addition, Hoehn and Yahr stage [15], levodopa equivalent daily dose (LEDD), and functional oral intake score (FOIS) [16] were determined before the beginning of LCIG and in December 2018. In the subgroup of patients who received FEES before and after the implementation of LCIG, these parameters were determined at the time of the FEES examinations.

### 2.3. Dysphagia Assessment

**Figure 1:**
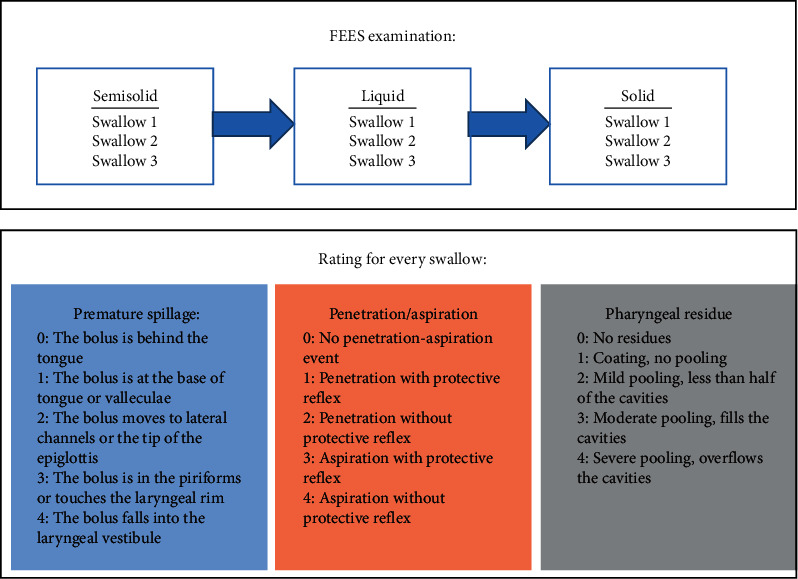
Illustration of the FEES examination protocol and the rating of the FEES videos.

Following our local guidelines, all PD patients with Hoehn and Yahr stage ≥2 regularly received a clinical dysphagia assessment by a trained speech-language pathologist. In those with clinical signs for PDrPD or in unclear cases, a FEES was conducted by a speech-language pathologist together with a trained neurologist. FEES in PD patients was performed following a stepwise protocol with testing of three different food consistencies in the following order: three trials of 8 ml of green jelly (semisolid), three trials of 5 ml of blue-dyed liquid, and three trials of white bread (solid) with a size of approximately 3 cm × 3 cm × 0.5 cm [10]. The consistencies according to the framework of the International Dysphagia Diet Standardization Initiative (IDDSI) were level 0 for liquid, level 4 for semisolid, and level 7 for solid [17]. FEES examination was regularly performed in the clinical “on-state,” and videos were stored on the hard drive for later review. The examination protocol is illustrated in Figure 1.

### 2.4. Rating of FEES Videos

For this study, the videos were systematically reassessed to determine the extent of PDrPD using the FEES-L-dopa-score [10]: The scoring evaluated three parameters of swallowing function: (1) premature bolus spillage, (2) penetration and aspiration, and (3) residue in the pharynx. Each parameter was rated on a scale from 0 (normal) to 4 (severe impairment) for every trial and each food consistency, contributing to an overall cumulative score of a maximum of 108. The scoring is further illustrated in Figure 1. This score was validated to detect clinically relevant changes of PDrPD due to dopaminergic medication [10]. The scores before and after beginning of LCIG therapy were determined. Further, the swallowing parameters of premature bolus spillage, penetration/aspiration, and pharyngeal residue were determined separately according to the respective score. In addition, the average white-out duration of the swallowing trials with solid consistencies was determined before and after beginning of LCIG. At the beginning of the pharyngeal phase of swallowing, the increased intrapharyngeal pressure causes the tip of the endoscope to be pressed against the pharyngeal wall. This leads to a white superimposition by reflection of the light from the distal end of the endoscope [18]. The beginning of the white-out was defined as the first frame in which the laryngeal structures were not visible due to white superimposition. Conversely, the end of the white-out was defined as the first frame in which all laryngeal structures were visible again [19, 20].

### 2.5. Statistical Analysis

Descriptive statistics were applied to quantify the demographic patient characteristics and the obtained clinical parameters. The data are presented as frequencies for categorical variables and mean values ± standard deviation (SD) for metric variables. Descriptive statistics are presented for the entire cohort as well as for the subgroup who received both FEES before and after the start of LCIG therapy.

In this subgroup, the FEES-L-dopa-score and its subdomains, the white-out duration of the swallowing trials with solid consistencies, and the LEDD before and after beginning of LCIG therapy were compared using the *t*-test for paired samples. The Hoehn and Yahr scale and FOIS scale before and after beginning of LCIG therapy were compared using the Wilcoxon signed rank test. Due to multiple testing (8 tests), the *p* value was adjusted and considered statistically significant at 0.05/8 = 0.006.

## 3. Results

### 3.1. Study Cohort

Forty-five patients who started LCIG therapy within the defined period fulfilled the initial inclusion criteria. Eleven of the 45 patients (24.4%) had PDrPD and had received an on-state FEES examination both before and after starting LCIG therapy. Descriptive statistics for the total cohort as well as for the subgroup with follow-up FEES examinations are shown in Table 1.

### 3.2. FEES Results

Descriptive statistics of the FEES-L-dopa-score and its subdomains, white-out duration, LEDD, Hoehn and Yahr scale, and FOIS scale as well as the *p* value for comparison before and after beginning of LCIG are shown in Table 2. The mean FEES-L-dopa score was lower after starting the LCIG therapy compared to before the treatment (14.9 vs. 13.0), but this difference was not statistically significant (*p*=0.312, *t* = 1.066). However, premature bolus spillage (*p*=0.002, *t* = 4.249) and white-out duration (*p*=0.002, *t* = 4.067) significantly decreased after implementation of LCIG.

## 4. Discussion

Premature bolus spillage and pharyngeal transit time (white-out duration) were significantly reduced after the beginning of LCIG therapy. These results suggest that LCIG affects PDrPD and may improve some swallowing pathologies. This effect on PDrPD was observed when comparing clinical “on-state” before and after starting LCIG treatment. Previous studies with instrumental evaluation have shown that PDrPD may be partly L-dopa responsive by comparing the “on-state” swallowing function after acute levodopa challenge with the “off-state” condition [10, 21]. The results of our study support the finding of an uncontrolled observational study in which up to 60% of PD patients subjectively experienced an improvement of swallowing function and dysphagia during LCIG therapy. [22].

The mechanism of LCIG affecting dysphagia is presumably similar to the mechanism of LCIG affecting motor function: With regards to motor symptoms, LCIG therapy not only prolongs the daily duration of the “on-state,” but also leads to further clinical improvement during the “on-state” [6, 23]. In this study, the average LEDD significantly increased after the start of LCIG therapy. A higher dopaminergic dose in combination with continuous application and less-fluctuating plasma levels could have led to an improvement of the dopamine-sensitive component of PDrPD, e.g., premature bolus spillage. The results of this study therefore indicate that LCIG may improve PDrPD if it is L-dopa responsive in principle. Thus, the FEES-L-dopa test might be a useful tool for estimating L-dopa responsiveness of PDrPD in individual patients [21].

PD patients may exhibit oropharyngeal bradykinesia [24–28]. Bradykinesia refers to the slowness of movement and is pathophysiologically attributed to a dysfunctional interaction between the basal ganglia and cortical areas involved in movement initiation and control [29, 30]. Similar to other motor functions, basal ganglia are activated during swallowing in neuroimaging studies and thus assumed to be part of a supranuclear network for initiating and controlling swallowing movements [31]. Oropharyngeal bradykinesia can lead to dysphagia symptoms such as decreased oral bolus control, prolonged transit times during swallowing, premature bolus spillage, drooling of saliva, delayed laryngeal vestibule closure, or aspiration [27, 28, 32]. The fact that white-out duration after beginning of LCIG significantly decreased in our study indicates that LCIG improves pharyngeal bradykinesia and thus shortens pharyngeal transit time due to faster pharyngeal movement. White-out duration is one of the most constant parameters in swallowing physiology, and its assessment shows an excellent interrater reliability [20, 33]. The beginning of white-out strongly corresponds to the hyoid elevation which defines the beginning of the pharyngeal phase of swallowing [20]. Therefore, white-out duration can be considered as a parameter for pharyngeal transit time [20]. The average white-out duration in this study before (984 ms) and after (699 ms) the beginning of LCIG was higher compared to the previously published average duration of 675 ms in healthy older subjects (65–74 years) when swallowing solid consistencies [20]. This also suggests the presence of pharyngeal bradykinesia in PD patients compared to healthy individuals.

Premature bolus spillage is one of the most common swallowing abnormalities in PD [10, 34]. In line with the results of our study, it has previously been shown to be a dopamine-sensitive pathology that improves after acute levodopa application [10]. A possible explanation for the reduction of premature bolus spillage could be that L-dopa application leads to an earlier triggering of the swallowing reflex [35].

It is important to note that PDrPD does not respond favorably to dopaminergic medication in all patients but only in approximately 50% [10, 21]. Therefore, also nondopaminergic mechanisms are likely to be involved in the pathophysiology of PDrPD. These include peripheral sensory impairment [36, 37], decreased substance P concentration [38], and consecutively reduced cough and protective reflexes [39], as well as cortical mechanisms [40]. The individual impact of these different influencing factors has hardly been investigated. So far it is unknown to what extent modulatory effects of dopaminergic and nondopaminergic mechanisms interact with each other. In our study, LCIG did not significantly influence global swallowing function (evaluated with the FEES-L-dopa-score). It therefore remains unclear whether LCIG has a positive effect on PDrPD in general. In patient groups with predominantly nondopaminergic dysphagia mechanisms or other swallowing pathologies than premature spillage and pharyngeal bradykinesia, LCIG may not have a positive effect or may even worsen PDrPD.

There are several limitations that must be considered when interpreting this study. The retrospective design, the small sample size, and the lack of a control group limited the statistical analysis or could have led to a selection bias. The FEES examinations did not take place in defined intervals to the beginning of LCIG but were variable which limits comparison. In addition to LCIG therapy, the cohort also included various other forms of therapy, for example, all patients with PDrPD were recommended speech and language therapy. The relationship between swallowing and motor function could not be analyzed as the motor function was not assessed. Due to the retrospective design and the small sample size, no valid statement was possible about relevant outcome parameters such as mortality, pneumonia rate, or weight progression. Future prospective studies are therefore mandatory to follow-up on these pilot results to determine the general effect of LCIG on global swallowing function and to determine which factors predict a good response of PDrPD to LCIG treatment in patients in the advanced stage of PD.

## 5. Conclusions

PDrPD responds to L-dopa application in about half of the patients. LCIG may therefore affect PDrPD similar to the mechanism of LCIG affecting motor function via a more stable plasma level of L-dopa. The results of our study indicate that LCIG might reduce premature bolus spillage and improve pharyngeal bradykinesia. Future studies with larger sample sizes are required to follow-up on these pilot results and identify which factors predict a good response of PDrPD to LCIG treatment.

## Figures and Tables

**Table 1:** Descriptive statistics for the total cohort as well as for the subgroup with follow-up FEES examinations before and after beginning with levodopa-carbidopa intestinal gel (LCIG).

*Gender men, n (%)*	
Total cohort	32 (71.1)
Subgroup	8 (72.7)

*Mean age,* *±* *SD*	
Total cohort	73.3 ± 8.2
Subgroup	74.6 ± 4.4

*Mean time of disease in years* *±* *SD*	
Total cohort	16.0 ± 6.5
Subgroup	15.2 ± 6.6

*Mean time of LCIG in month* *±* *SD*	
Total cohort	27.0 ± 22.3
Subgroup	28.4 ± 18.8

*Dysphagia in FEES, n (%)*	
Total cohort	28 (62.2)
Subgroup	11 (100.0)

*Dietary supplement, n (%)*	
Total cohort	9 (20.0)
Subgroup	2 (18.2)

*Infections, n (%)*	
Total cohort	11 (24.4)
Subgroup	2 (18.2)

*Pneumonia, n (%)*	
Total cohort	5 (11.1)
Subgroup	1 (9.1)

*Discontinuation LCIG, n (%)*	
Total cohort	3 (6.7)
Subgroup	1 (9.1)

*Complication total LCIG, n (%)*	
Total cohort	18 (40.0)
Subgroup	4 (36.4)

*Dislocation LCIG, n (%)*	
Total cohort	16 (35.6)
Subgroup	3 (27.3)

*Death, n (%)*	
Total cohort	3 (6.7)
Subgroup	1 (9.1)

*Dementia, n (%)*	
Total cohort	22 (48.9)
Subgroup	7 (63.6)

**Table 2:** Descriptive statistics of the FEES-L-dopa-score and its subdomains, white-out duration, levodopa equivalent daily dose (LEDD), Hoehn and Yahr scale, and functional oral intake scale (FOIS), as well as the *p* value of the comparison before and after beginning of levodopa-carbidopa intestinal gel (LCIG) in the total cohort and the subgroup of patients with follow-up FEES before and after beginning with LCIG.

	Before LCIG	After LCIG	*p* value
*Mean FEES-L-dopa-score* *±* *SD*			
Subgroup	14.9 ± 7.3	13.0 ± 6.9	0.312

*Mean score premature spillage* *±* *SD*			
Subgroup	5.4 ± 1.1	3.6 ± 1.0	^*∗*^ ** 0.002**

*Mean score penetration/aspiration* *±* *SD*			
Subgroup	0.3 ± 0.9	0.5 ± 1.0	0.690

*Mean score residue* *±* *SD*			
Subgroup	9.3 ± 5.9	8.9 ± 6.0	0.795

*Mean white-out duration in ms* *±* *SD*			
Subgroup	984 ± 228	699 ± 131	^*∗*^ ** 0.002**

*Mean Hoehn and Yahr* *±* *SD*			
Total cohort	3.8 ± 0.7	3.5 ± 0.7	0.078
Subgroup	3.6 ± 0.5	3.8 ± 0.6	0.317

*Mean LEDD* *±* *SD*			
Total cohort	1182.9 ± 339.3	1684.2 ± 468.2	^*∗*^ ** <0.001**
Subgroup	1246.1 ± 371.9	2062.0 ± 379.2	^*∗*^ ** <0.001**

*Mean FOIS* *±* *SD*			
Total cohort	6.6 ± 1.1	6.6 ± 1.1	0.832
Subgroup	6.4 ± 0.9	6.6 ± 0.5	0.414

## Data Availability

The patient history and patient diagnostic data used to support the findings of this study have not been made available because they are restricted by the local ethics committee of the University of Muenster to protect the privacy of patients and may only be made available to researchers participating in this study.

## References

[1] Ahlskog J. E., Muenter M. D. (2001). Frequency of levodopa-related dyskinesias and motor fluctuations as estimated from the cumulative literature. *Movement Disorders*.

[2] Schrag A., Quinn N. (2000). Dyskinesias and motor fluctuations in parkinson’s disease. A community-based study. *Brain: A Journal of Neurology*.

[3] Othman A. A., Rosebraugh M., Chatamra K., Locke C., Dutta S. (2017). Levodopa-carbidopa intestinal gel pharmacokinetics: lower variability than oral levodopa-carbidopa. *Journal of Parkinson’s Disease*.

[4] Nyholm D., Askmark H., Gomes–Trolin C. (2003). Optimizing levodopa pharmacokinetics: intestinal infusion versus oral sustained-release tablets. *Clinical Neuropharmacology*.

[5] Olanow C. W., Kieburtz K., Odin P. (2014). Continuous intrajejunal infusion of levodopa-carbidopa intestinal gel for patients with advanced parkinson’s disease: a randomised, controlled, double-blind, double-dummy study. *The Lancet Neurology*.

[6] Slevin J. T., Fernandez H. H., Zadikoff C. (2015). Long-term safety and maintenance of efficacy of levodopa-carbidopa intestinal gel: an open-label extension of the double-blind pivotal study in advanced parkinson’s disease patients. *Journal of Parkinson’s Disease*.

[7] Takizawa C., Gemmell E., Kenworthy J., Speyer R. (2016). A systematic review of the prevalence of oropharyngeal dysphagia in stroke, parkinson’s disease, alzheimer’s disease, head injury, and pneumonia. *Dysphagia*.

[8] Fasano A., Visanji N. P., Liu L. W. C., Lang A. E., Pfeiffer R. F. (2015). Gastrointestinal dysfunction in parkinson’s disease. *The Lancet Neurology*.

[9] Suttrup I., Warnecke T. (2016). Dysphagia in parkinson’s Disease. *Dysphagia*.

[10] Warnecke T., Suttrup I., Schröder J. B. (2016). Levodopa responsiveness of dysphagia in advanced parkinson’s disease and reliability testing of the FEES-levodopa-test. *Parkinsonism & Related Disorders*.

[11] Pflug C., Bihler M., Emich K. (2018). Critical dysphagia is common in parkinson disease and occurs even in early stages: a prospective cohort study. *Dysphagia*.

[12] Dziewas R., auf dem Brinke M., Birkmann U. (2019). Safety and clinical impact of FEES–results of the FEES-registry. *Neurological Research and Practice*.

[13] Hughes A. J., Daniel S. E., Kilford L., Lees A. J. (1992). Accuracy of clinical diagnosis of idiopathic parkinson’s disease: a clinico-pathological study of 100 cases. *Journal of Neurology, Neurosurgery & Psychiatry*.

[14] Neubauer P. D., Rademaker A. W., Leder S. B. (2015). The Yale pharyngeal residue severity rating scale: an anatomically defined and image-based tool. *Dysphagia*.

[15] Hoehn M. M., Yahr M. D. (1967). Parkinsonism: onset, progression, and mortality. *Neurology*.

[16] Crary M. A., Mann G. D. C., Groher M. E. (2005). Initial psychometric assessment of a functional oral intake scale for dysphagia in stroke patients. *Archives of Physical Medicine and Rehabilitation*.

[17] Cichero J. A. Y., Lam P., Steele C. M. (2017). Development of international terminology and definitions for texture-modified foods and thickened fluids used in dysphagia management: the IDDSI framework. *Dysphagia*.

[18] Langmore S. E. (2006). *Endoscopic Evaluation of Oral and Pharyngeal Phases of Swallowing*.

[19] Warnecke T., Oelenberg S., Teismann I. (2009). Dysphagia in X-linked bulbospinal muscular atrophy (kennedy disease). *Neuromuscular Disorders*.

[20] Mozzanica F., Lorusso R., Robotti C. (2019). Effect of age, sex, bolus volume, and bolus consistency on whiteout duration in healthy subjects during FEES. *Dysphagia*.

[21] Warnecke T., Oelenberg S., Teismann I. (2010). Endoscopic characteristics and levodopa responsiveness of swallowing function in progressive supranuclear palsy. *Movement Disorders*.

[22] Devos D., Lingua::EN::Titlecase fnm (2009). Patient profile, indications, efficacy and safety of duodenal levodopa infusion in advanced parkinson’s disease. *Movement Disorders*.

[23] Antonini A., Yegin A., Preda C., Bergmann L., Poewe W. (2015). Global long-term study on motor and non-motor symptoms and safety of levodopa-carbidopa intestinal gel in routine care of advanced parkinson’s disease patients; 12 month interim outcomes. *Parkinsonism & Related Disorders*.

[24] Pfeiffer R. F. (2011). Gastrointestinal dysfunction in parkinson’s disease. *Parkinsonism & Related Disorders*.

[25] Gates J., Hartnell G. G., Gramigna G. D. (2006). Videofluoroscopy and swallowing studies for neurologic disease: a primer. *Radiographics: a review publication of the Radiological Society of North America, Inc*.

[26] Umemoto G., Tsuboi Y., Kitashima A., Furuya H., Kikuta T. (2011). Impaired food transportation in parkinson’s disease related to lingual bradykinesia. *Dysphagia*.

[27] Ellerston J. K., Heller A. C., Houtz D. R., Kendall K. A. (2016). Quantitative measures of swallowing deficits in patients with parkinson’s disease. *Annals of Otology, Rhinology & Laryngology*.

[28] Wakasugi Y., Yamamoto T., Oda C., Murata M., Tohara H., Minakuchi S. (2017). Effect of an impaired oral stage on swallowing in patients with parkinson’s disease. *Journal of Oral Rehabilitation*.

[29] Berardelli A., Rothwell J. C., Thompson P. D. (2001). Pathophysiology of bradykinesia in parkinson’s disease. *Brain: A Journal of Neurology*.

[30] Wichmann T. (2019). Changing views of the pathophysiology of parkinsonism. *Movement Disorders: Official Journal of the Movement Disorder Society*.

[31] Leopold N. A., Daniels S. K. (2010). Supranuclear control of swallowing. *Dysphagia*.

[32] Karakoc M., Yon M. I., Cakmakli G. Y. (2016). Pathophysiology underlying drooling in parkinson’s disease: oropharyngeal bradykinesia. *Neurological Sciences: Official Journal of the Italian Neurological Society and of the Italian Society of Clinical Neurophysiology*.

[33] Logemann J. A., Rademaker A. W., Pauloski B. R., Ohmae Y., Kahrilas P. J. (1999). Interobserver agreement on normal swallowing physiology as viewed by videoendoscopy. *Folia Phoniatrica et Logopaedica*.

[34] Manor Y., Mootanah R., Freud D., Giladi N., Cohen J. T. (2013). Video-assisted swallowing therapy for patients with parkinson’s disease. *Parkinsonism & Related Disorders*.

[35] Kobayashi H., Nakagawa T., Sekizawa K. (1996). Levodopa and swallowing reflex. *Lancet (London, England)*.

[36] Mu L., Sobotka S., Chen J. (2013). Parkinson disease affects peripheral sensory nerves in the pharynx. *Journal of Neuropathology & Experimental Neurology*.

[37] Labeit B., Muhle P., Ogawa M. (2019). FEES-based assessment of pharyngeal hypesthesia-Proposal and validation of a new test procedure. *Neurogastroenterology and Motility: The Official Journal of the European Gastrointestinal Motility Society*.

[38] Schroder J. B., Marian T., Claus I. (2019). Substance P saliva reduction predicts pharyngeal dysphagia in parkinson’s disease. *Frontiers in Neurology*.

[39] Troche M. S., Brandimore A. E., Okun M. S., Davenport P. W., Hegland K. W. (2014). Decreased cough sensitivity and aspiration in parkinson disease. *Chest*.

[40] Suntrup S., Teismann I., Bejer J. (2013). Evidence for adaptive cortical changes in swallowing in parkinson’s disease. *Brain: A Journal of Neurology*.

